# Phylogeny, character evolution and historical biogeography of Scurrulinae (Loranthaceae): new insights into the circumscription of the genus *Taxillus*

**DOI:** 10.1186/s12870-024-05126-0

**Published:** 2024-05-22

**Authors:** Chi Toan Le, Limin Lu, Van Du Nguyen, Zhiduan Chen, Wyckliffe Omondi Omollo, Bing Liu

**Affiliations:** 1 State Key Laboratory of Plant Diversity and Specialty Crops, Institute of Botany, Chinese Academy of Sciences, Beijing, 100093 China; 2 Hanoi Pedagogical University 2, 32 Nguyen Van Linh, Xuanhoa, Phucyen, Vinhphuc Vietnam; 3 Institute of Ecology and Biological Resources (IEBR), Vietnam Academy of Science and Technology (VAST), Hoang Quoc Viet, Cau Giay, Hanoi, Vietnam; 4 Graduate University of Science and Technology, Vietnam Academy of Science and Technology, Hoang Quoc Viet, Cau Giay, Hanoi, Vietnam; 5 University of Chinese Academy of Sciences, Beijing, 100049 China

**Keywords:** Phylogeny, Historical biogeography, Scurrulinae, *Phyllodesmis*, Africa, Taxonomy, Asia

## Abstract

**Background:**

Exploring the relationship between parasitic plants and answering taxonomic questions is still challenging. The subtribe Scurrulinae (Loranthaceae), which has a wide distribution in Asia and Africa, provides an excellent example to illuminate this scenario. Using a comprehensive taxon sampling of the subtribe, this study focuses on infer the phylogenetic relationships within Scurrulinae, investigate the phylogeny and biogeography of the subtribe, and establish a phylogenetically-based classification incorporating both molecular and morphological evidence. We conducted phylogenetic, historical biogeography, and ancestral character state reconstruction analyses of Scurrulinae based on the sequences of six DNA regions from 89 individuals to represent all five tribes of the Loranthaceae and the dataset from eleven morphological characters.

**Results:**

The results strongly support the non-monophyletic of Scurrulinae, with *Phyllodesmis* recognized as a separate genus from its allies *Taxillus* and *Scurrula* based on the results from molecular data and morphological character reconstruction. The mistletoe Scurrulinae originated in Asia during the Oligocene. Scurrulinae was inferred to have been widespread in Asia but did not disperse to other areas. The African species of *Taxillus*, *T. wiensii*, was confirmed to have originated in Africa from African Loranthaceae ca. 17 Ma, and evolved independently from Asian members of *Taxillus*.

**Conclusions:**

This study based on comprehensive taxon sampling of the subtribe Scurrulinae, strongly supports the relationship between genera. The taxonomic treatment for *Phyllodesmis* was provided. The historical biogeography of mistletoe Scurrulinae was determined with origin in Asia during the Oligocene. *Taxillus* and *Scurrula* diverged during the climatic optimum in the middle Miocene. *Taxillus wiensii* originated in Africa from African Loranthaceae, and is an independent lineage from the Asian species of *Taxillus*. Diversification of Scurrulinae and the development of endemic species in Asia may have been supported by the fast-changing climate, including cooling, drying, and the progressive uplift of the high mountains in central Asia, especially during the late Pliocene and Pleistocene.

**Supplementary Information:**

The online version contains supplementary material available at 10.1186/s12870-024-05126-0.

## Background

Loranthaceae is the largest mistletoe family within the order Santalales, comprising approximately 76 genera and over 1000 species. Species of the family are mainly distributed in tropical and subtropical regions of America, Africa, Asia, and Australia, with a few extending to the temperate zones in Europe and East Asia [1–5]. The aerial parasitic species of Loranthaceae exhibit unique morphology with a haustorium connection structure that adds to the family’s diversity worldwide. The subtribe Scurrulinae, comprising *Scurrula* (ca. 50 spp.) and *Taxillus* (ca. 35 spp.), was established by Nickrent et al. [2] and recognized by Kuijt [4], Liu et al. [5] and Su et al. [6]. Most members of Scurrulinae are distributed in Asia, except for *Taxillus wiensii* Polhill, the sole species in Africa [7, 8].

The subtribe Scurrulinae has received limited taxonomic attention except for the Chinese flora. Kiu [9] identified 26 Chinese species of the subtribe in Flora Reipublicae Popularis Sinicae, belonging to *Scurrula* and *Taxillus*, respectively. The genus *Scurrula* was divided into two sections: section *Cichlanthus* which includes six species and section *Scurrula* with five species [9]. The fruit base tapers into stalk in section *Cichlanthus*, while the fruit base does not form stalk in section *Scurrula*. The genus *Taxillus* was divided into three sections. The section *Phyllodesmis*, which includes *Taxillus delavayi*, *T. kaempferi*, and *T. caloreas*, is characterized by the fascicled leaves on short shoots and glabrous corolla (vs. non-fascicled leaves and hairy corolla in the other two sections). The section *Lancilobi* includes seven species and bears flowers with lanceolate corolla lobe, while the section *Spathulilobi* includes five species and the shape of corolla lobe is spathulate [9]. Kiu and Gilbert [8] recognized 28 species of Scurrulinae in Flora of China, including 10 species for *Scurrula* and 18 species for *Taxillus*. The authors suggested that *Scurrula* and *Taxillus* are generally similar in morphology such as opposite or subopposite leaves, axillary inflorescences, bisexual, 4-merous and zygomorphic flowers. Nonetheless, differences in calyx and fruit shape are important to distinguish the two genera, where *Scurrula* has stipitate, obovoid or clavate fruits and *Taxillus* has non-stipitate, ovoid or ellipsoid fruits.

The genus *Phyllodesmis* was established by Tieghem [10] with three species *P. delavayi*, *P. paucifolia* and *P. coriacea*. However, the genus was later reduced as a section of *Taxillus* [9] or synonym of *Taxillus* [8]. The molecular phylogenetic relationships of the three genera remain unclear. Therefore, it is important to test species delimitation in *Taxillus* and *Phyllodesmis* with a well-resolved phylogenetic framework based on intense taxon sampling.

Phylogenetic studies have shown that Scurrulinae is closely related to Dendrophthoinae and African Loranthaceae [3, 5, 6, 11]. The African Loranthaceae includes all the members native to continental Africa and Madagascar. Besides of the genera assigned to the subtribes Emelianthinae and Tapinanthinae sensu Nickrent et al. [2], there are also some species of *Helixanthera* and *Taxillus* distributed in Africa [4, 7]. In particular, Vidal-Russell and Nickrent [3] found that Scurrulinae was the sister group of Dendrophthoinae and African Loranthaceae, but only four members of Scurrulinae were sampled. Liu et al. [5] investigated the phylogenetic relationship and biogeography of the family Loranthaceae, which included 11 samples of Scurrulinae. This study supported that Scurrulinae including *Scurrula* and *Taxillus* formed a well-supported monophyletic group, which was estimated to originate in Asia during the early Oligocene. However, the phylogeny and classification of the subtribe were not thoroughly discussed.

The morphological characters are significant to understand generic circumscription, species delimitation, and morphological evolution [12]. Furthermore, morphological variation of some characters such as trichomes of leaf or stem, corolla lobe number and fruit shape deserve further evaluation to delimit species boundaries of Scurrulinae and related taxa. In addition, the inclusion of morphological analyses is also needed to fully unravel the relationships among the species of these two genera.

To resolve the phylogenetic position of Scurrulinae and relationships within this group, studies with a comprehensive taxon sampling to focus on the phylogenetic relationship, classification, and historical biogeography of Scurrulinae are required. The established phylogenetic framework should facilitate further collections-based integrative studies involving biogeographic and phylogenetic analyses of Scurrulinae and its close allies. Thus, the present study aims to (1) investigate the phylogenetic position of the subtribe Scurrulinae and infer the phylogenetic relationships within Scurrulinae; (2) reconstruct the biogeographical history of Scurrulinae and determine the origin of lineages within this subtribe; and (3) establish a phylogenetically-based classification incorporating both molecular and morphological evidence.

## Materials and methods

### Sampling, DNA extraction, and sequencing

We collected a total of 89 individuals to ensure representation of all five tribes of the Loranthaceae, with 52 of the 89 individuals belonging to the subtribe Scurrulinae. The molecular matrix was constructed using the nuclear small-subunit ribosomal DNA (SSU rDNA), large-subunit ribosomal DNA (LSU rDNA), ribosomal internal transcribed spacer (ITS) and three chloroplast including *rbc*L, *mat*K and *trn*L-F. We downloaded sequences from NCBI and generated sequences from our own collections. The voucher information and GenBank accession numbers are listed in Table S1.

We extracted Genomic DNA of all samples by using the dried leaf tissues following the CTAB procedure [13]. The primers used for PCR and sequencing in this study were referred from Vidal-Russell and Nickrent [3, 14], Wilson and Calvin [15] and Taberlet et al. [16] (Applied Biosystems, USA). We used Geneious v.8.0.5 [17] to align the sequences in this study.

### Phylogenetic analyses

The two methods maximum likelihood (ML) and Bayesian inference (BI) were used to perform phylogenetic analyses of Scurrulinae. We partitioned the combined of Scurrulinae dataset into six subsets corresponding to the six DNA regions with each partition by the Akaike Information Criterion (AIC) as implemented in jModelTest v.2.1.6 [18], which was accessed through the CIPRES Science Gateway [19].

The ML analysis was performed in RAxML v.8.2.12 [20, 21] with the best substitution models of each partition by running 1000 bootstrap replicates. We conducted the BI in MrBayes v.3.1.2 [22] on the CIPRES Science Gateway Portal [19] applying the same substitution models as in the ML analysis. In the BI analysis, the Markov chain Monte Carlo (MCMC) algorithm was set running for 10 million generations with a total of four chains, starting from a random tree, and trees were sampled every 1000 generations. The effective sample sizes (ESSs) were checked to ensure all relevant parameters are higher than 200 by using the program Tracer v.1.6 [23]. We discarded the first 25% of sampled generations, then use the remaining trees to obtain the majority-rule consensus tree and Bayesian posterior probabilities (PP). The clades with BS ≥ 70% and PP ≥ 0.95 (95%) are considered to be strongly supported.

### Ancestral character state reconstruction

We chose eleven morphological characters, including seven binaries and four multistate ones (Table 1) for ancestral character state reconstruction. The morphological traits used to reconstruct the character matrix were recognized through field observations, examining specimens and consulting published literature [4, 6–8, 24]. Specimens from the Institute of Botany, Chinese Academy of Sciences, Beijing, China herbarium (PE), VNU University of Science, Hanoi, Vietnam (HNU) and Institute of Ecology and Biological Resources, Hanoi, Vietnam (HN) were also carefully examined. The herbarium acronyms follow the data of Index Herbariorum (http://sweetgum.nybg.org/ih/). We detailed the morphological characters and the coding of character states in Table S3. In addition, the descriptions from the Flora of China account [8] have been assessed and used as the basis for an expanded and detailed description of a single genus and species.

**Table 1:** List of the 11 morphological characters and character states scored for the ancestral character state reconstruction of Scurrulinae

1.	Morphological characters and states
	Young stems tomentum: glabrous (0); trichomes present (1).
2.	Leaf placement: opposite or subopposite (0); alternate (1).
3.	Leaf tomentum: glabrous (0); trichomes present (1).
4.	Inflorescence type: umbel (0); racemes (1); subumbels (2).
5.	Corolla tomentum: glabrous (0); trichomes present (1).
6.	Corolla lobe number: 4 only (0); 5 only (1).
7.	Flower pedicel: absent (0); present (1).
8.	Bract shape: broad-triangular (0); narowly boat - shaped (1); ovate (2); triangular (3).
9.	Bract length: < 2 mm (0); >= 2 mm (1).
10.	Stigma: capitate to subcapitate (0); globose to subglobose (1).
11.	Fruit shape: ellipsoid or ovoid or subglobose (0); pyriform or clavate (1); cylindric (2).

The ancestral reconstruction analysis was conducted in Mesquite v.3.61 with the “Trace Character History” option and the ML approach using the Markov k-state one-parameter (Mk1) evolutionary model [25]. We reduced the taxon sampling in the molecular dataset by keeping only one individual per species to reconstruct the ancestral character. The topological framework is congruent with the trees in the phylogenetic analysis.

The observation of morphological characters was carried out from herbarium specimens and living plants in the field. Extended descriptions of these species have been made with reference to the relevant taxonomic literature [4, 8, 9].

### Divergence time estimation

To estimate the origin and divergence times of Scurrulinae within the Loranthaceae, we used the combined datasets from nuclear (ITS, LSU rDNA and SSU rDNA) and plastid (*rbc*L, *mat*K, *trn*LF) sequences for the dating analysis. This adjustment was made to match the markers used in previous phylogenetic studies from Santalales [3, 5, 6, 26].

The divergence time estimation of Scurrulinae was conducted by applying the uncorrelated lognormal Bayesian method in BEAST v.1.8.4 [27]. We partitioned the combined datasets based on six DNA regions using the “unlink substitution model” option, and on another hand, each partition of the combined dataset was run with a substitution model resulting from jModelTest v.2.1.6 [18]. All divergence time analyses were run using a Yule process tree prior, while, the four calibration points of dating analysis applied lognormal distribution option. The two separate Markov chain Monte Carlo was run for 150 million generations with samples taken every 1,500 generations.

We used Tracer v.1.6 [23] to check ESSs and ensure that all relevant parameters are higher than 200 and stationarity had been reached. After discarding the first 25% of trees as burn-in, TreeAnnotator v.1.8.0 [28] constructed a maximum credibility tree. Figtree v1.4.0 was used to illustrate the outcome [29].

Several palaeobotanical and palynological as well as historical biogeography studies were conducted on Santalales. Most of the Santalales fossils are pollen grains of Cretaceous and Tertiary periods [5, 14]. In this study, we selected one fossil calibration for the outgroups and three fossil calibrations for ingroups. The genus *Compositoipollenites* was recorded as fossil pollen of the *Misodendrum* from the middle Eocene [5, 30]. The pollen record exclusively consists of dispersed pollen grains allocated to the extinct genera *Sparsipollis* and *Compositoipollenites*, which have been recognized as members of Misodendraceae [30, 31]. The fossil pollen record of the family Misodendraceae was dated approximately 45 Ma during the Eocene in southern Patagonia and exists to the present [30]. Thus we constrained the crown age of Misodendraceae to 45 Ma (95% HPD: 41.2 Ma–48.6 Ma).

For the ingroups, the stem node of Loranthaceae was restricted to 70 Ma (95% HPD: 69.4 Ma–72.6 Ma) using the *Cranwellia* fossil [5, 31, 32]. The first occurrence of *Cranwellia* pollen was during the Campanian in Antarctica. Mildenhall [32] and Macphail et al. [31] suggested that *Cranwellia* pollen was recorded from Maastrichtian to early Pleistocene, and *Cranwellia* was assumed to be a member of Loranthaceae. We here consider that *Cranwellia* belongs to Loranthaceae, and used it to calibrate the stem node of Loranthaceae. The fossil pollen of the tribe Lorantheae identified to Changchang MT fossil as *Taxillus*, *Scurrula*, and *Amyema* [26]. Changchang MT is highly similar to the pollen of Scurrulinae (*Taxillus* and *Scurrula*) and Amyeminae (*Amyema*), thus it could be an early member (probably extinct) of the Lorantheae lineage related to the core Lorantheae. Changchang MT has been used to constrain the minimum age of the MRCA of Lorantheae [5, 26] during the late Eocene. We here accept Changchang MT as Lorantheae, and used it to calibrate its crown node. The fossil pollen Profen MT3 [26] was used to constrained the crown age of the tribe Elytrantheae to 39.6 Ma (95% HPD: 38 Ma–41.2 Ma). Profen MT3 represents some members of Elytrantheae, e.g. *Peraxilla tetrapetala* (L. f.) Tiegh., and occurred during the late Eocene [5, 26]. We thus used Profen MT3 to calibrate the crown node of Elytrantheae.

### Biogeographic reconstruction

BioGeoBEARS [33] and the Bayesian approach to dispersal-vicariance analysis (Bayes-DIVA [33]) were utilized to reconstruct the biogeographic history of Scurrulinae using the time tree obtained from BEAST.

The BioGeoBEARS was conducted in R [34]. The three likelihood-based models, including Dispersal-Extinction-Cladogenesis (DEC [35]), the likelihood version of dispersal–vicariance (DIVA [36]; hereafter DIVALIKE), and the likelihood version of BayArea model [37] (hereafter BAYAREALIKE) were used to analyze the biogeographic history of Scurrulinae. We used an additional “j” parameter (founder event/jump speciation) to evaluate if descendant lineages have a different region from the direct ancestor, the “j” parameter was added to all models [5, 33, 38]. We obtained a total of 6 models. Additionally, we calculated the number and kinds of biogeographical events by using the best-fit biogeographical model and biogeographical stochastic mapping (BSM) in ‘BioGeoBEARS’ [39]. According to the models, the biogeographical events were split into range expansions and founder events, vicariance, and within-area speciation events [40].

The dispersal-vicariance analysis of Bayesian was performed in RASP v.3.2 [41, 42]. Using the trees produced by BEAST, the Bayes-DIVA approach can increase phylogenetic certainty [41, 43]. We used 10,000 trees resulting from the dating and removed 25% of the trees to obtain a condensed tree as the final representative tree with the outgroups pruned.

Biogeographic area for species within the subtribe Scurrulinae were compiled from the distribution information described in the literatures and herbarium specimens (PE, K, P, HN) for extant Scurrulinae and their relatives [5, 7–9]. Although the subtribe Scurrulinae is distributed in Asia and Africa, this study defined four biogeographical areas following the distribution of extant Scurrulinae and outgroup species, as well as considering the definition of biogeographical areas in Liu et al. [5]: A = Asia; B = Australasia; C = Africa (including Arabian Peninsula); D = Americas. We did not define the Indian subcontinent as a separate biogeographical area because around 136 Ma [44], the subcontinent was drifting from Australia-Antarctica earlier than the origin of the Loranthaceae and Scurrulinae.

Madagascar includes only three genera of Loranthaceae, excluding Scurrulinae. Furthermore, Liu et al. [5] suggested that Madagascan Loranthaceae originated from Africa. Madagascar was thus not recognized as a biogeographical area in this study.

## Results

### Phylogenetic relationship

**Fig. 1:**
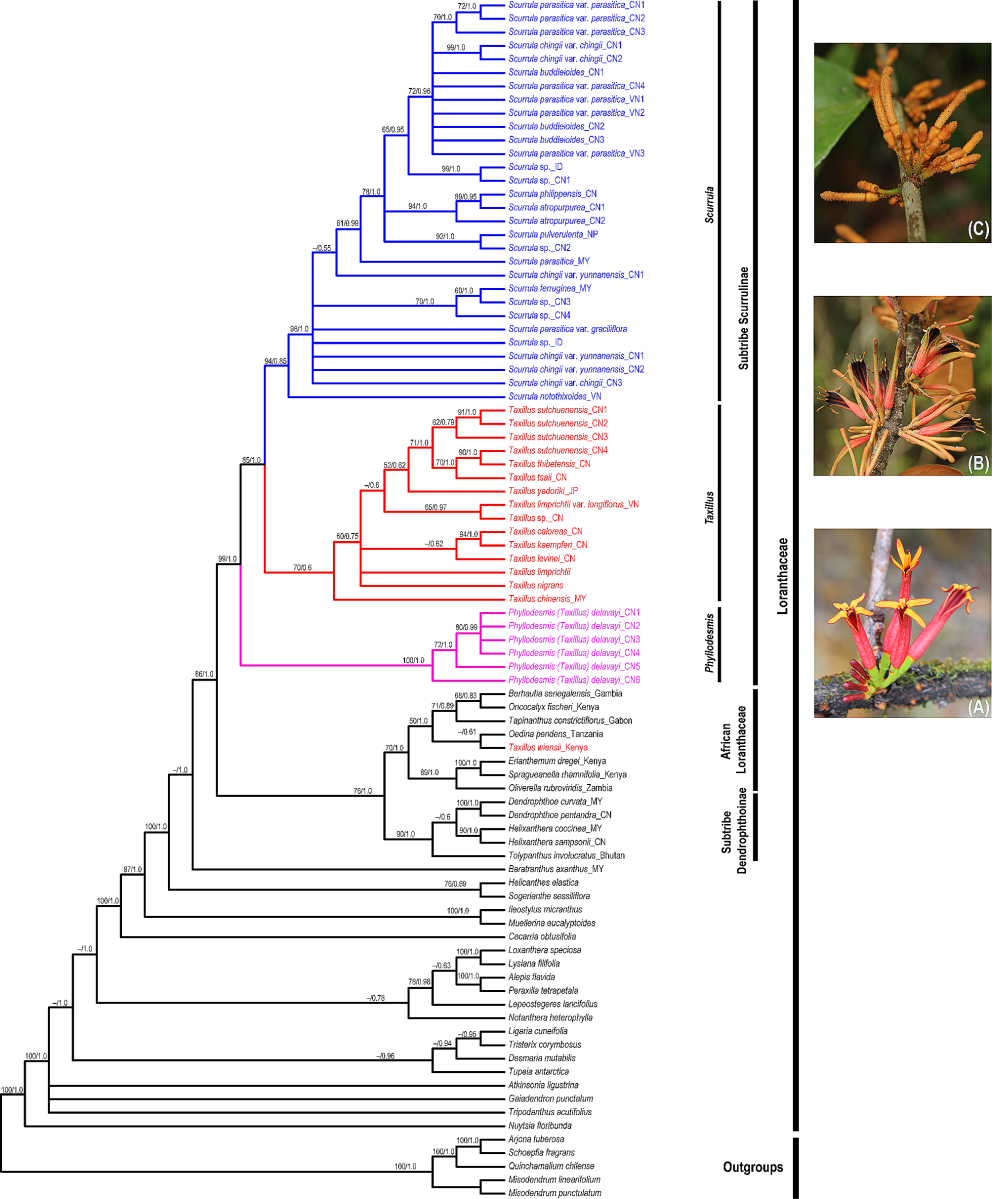
Majority rule consensus tree of Scurrulinae based on Bayesian inference of the combined dataset of six DNA regions (LSU rDNA, SSU rDNA, ITS, *mat*K, *rbc*L and *trn*L-F). ML bootstrap values and posterior probabilities (PP) of the BI analysis are presented above the branches. “–” indicates the support values less than 50%. **(A)**. *Phyllodesmis delavayi*; **(B)**. *Taxillus thibetensis;***(C)**. *Scurrula yunnanensis*. Photo credits: C. T. Le (**A**, **B**). Liu (**B**, **C**). Abbreviations: CN: China, VN: Vietnam, ID: Indonesia; MY: Malaysia, JP: Japan, NP: Nepal

We newly generated 164 sequences belonging to 46 individuals and downloaded 213 sequences of 51 individuals in this study, resulting in a matrix of 7,778 characters. Table S2 provides detailed information on each DNA region. In comparison to the combined dataset, phylogenetic trees based on six individual DNA regions and plastid partitions revealed relationships within the Scurrulinae with poorer resolution. The phylogenetic results based on the combined dataset from ML and BI analyses were highly congruent and we thus present the Bayesian tree with BS and PP values in Fig. 1.

Loranthaceae was supported as monophyletic, with *Nuytsia* as sister to the remaining genera (Fig. 1). The three root parasitic genera were placed in a basal clade of Loranthaceae.

Subtribe Scurrulinae was supported as non-monophyletic. Three close genera *Phyllodesmis*, *Taxillus*, and *Scurrula* placed in three distinct subclades with strongly supported (Fig. 1). The basal subclade including only *Phyllodesmis delavayi* was well supported as sister to the remaining two genera *Taxillus* and *Scurrula*. The genus *Taxillus* was found to be closely related to *Scurrula*, and our results showed that *Taxillus* was not monophyletic with one species *Taxillus wiensii* nested in the African Loranthaceae. Within the *Taxillus* clade, *T. chinensis* was weakly supported as sister to the remaining members (BS = 70%, PP = 0.6) (Fig. 1), while the positions of *T. limprichtii* and *T. nigrans* were unresolved. *T. kaempferi*, *T. caloreas*, and *T. levinei* formed a clade with low support, and the remaining species of *Taxillus* formed a clade. Our results supported the monophyly of *Scurrula*, with *S. notothixoides* as sister to the remaining species (Fig. 1). Within the genus *Scurrula*, relationships of some species were unresolved. *S. parasitica* was a complex species that was not monophyletic and appeared in some clades within *Scurrula*. On the other hand, *Taxillus wiensii* from Africa is placed within African Loranthaceae.

## Ancestral character state reconstruction

**Fig. 2:**
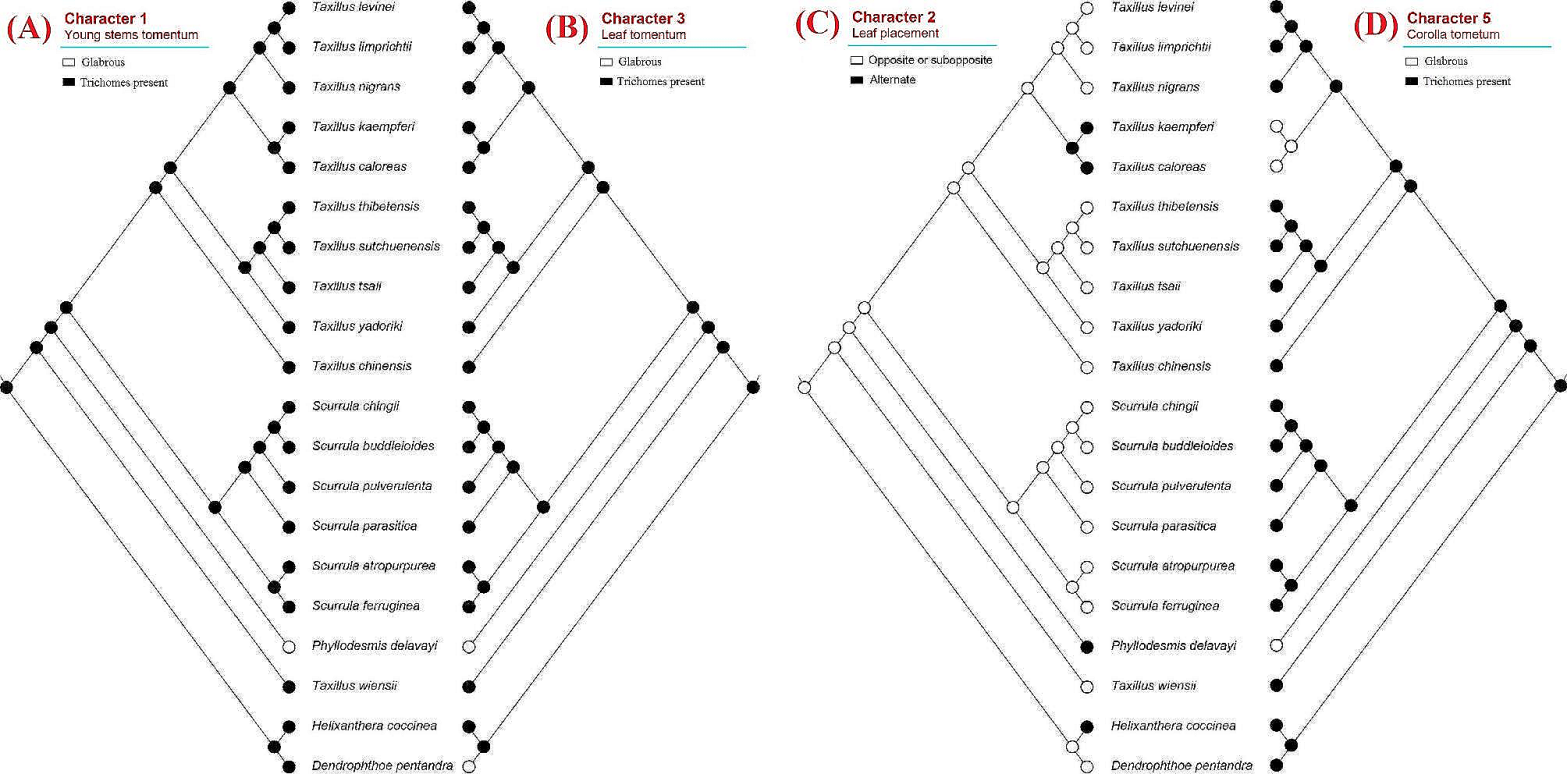
Character optimization of four morphological characters using the molecular phylogeny. **(A)** Young stems tomentum; **(B)** leaf tomentum; **(C)** leaf placement; **(D)** corolla tomentum

**Fig. 3:**
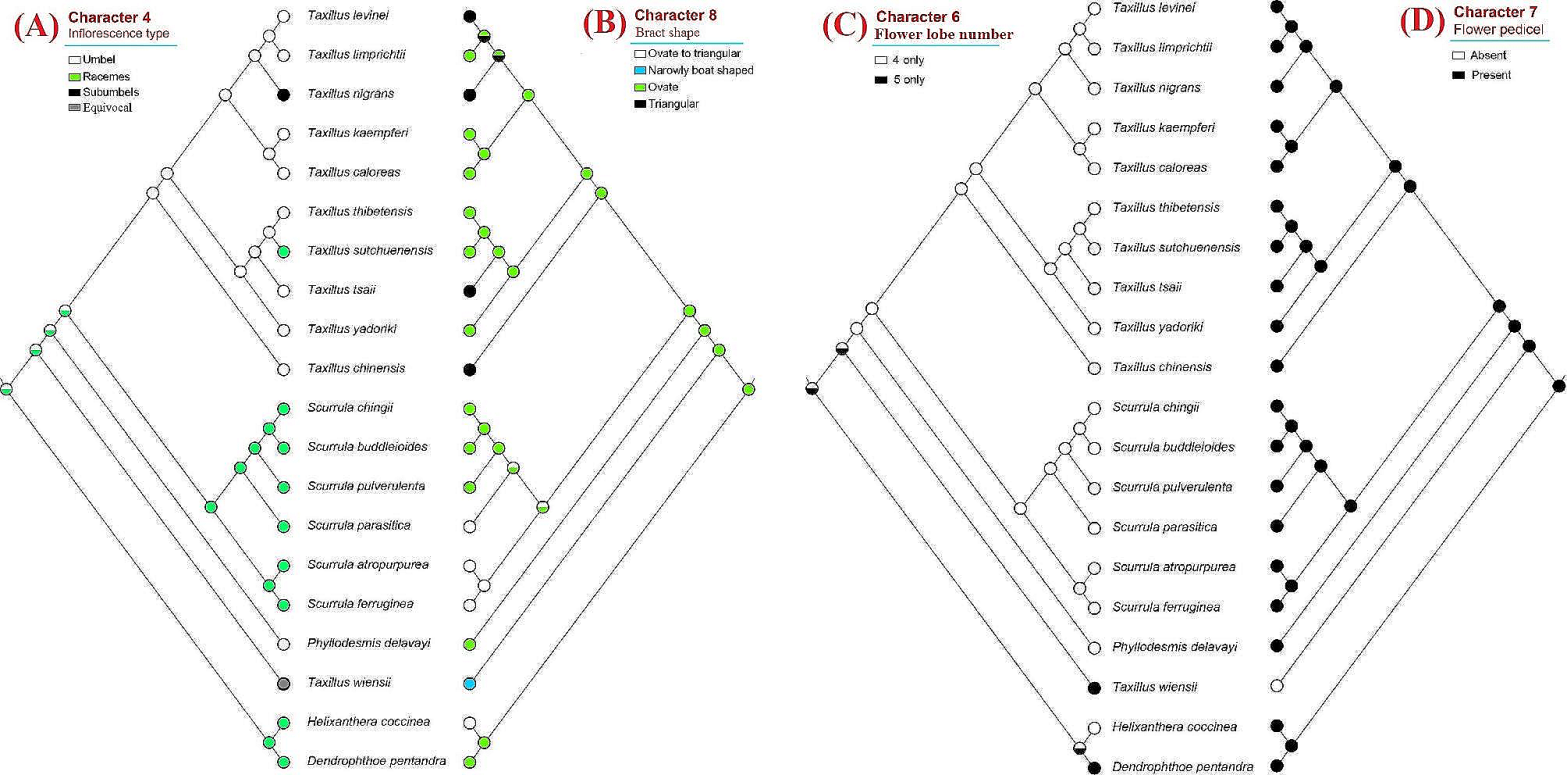
Character optimization of four morphological characters using the molecular phylogeny. **(A)** Inflorescence type; **(B)** bract; **(C)** corolla lobe number; **(D)** flower pedicel

**Fig. 4:**
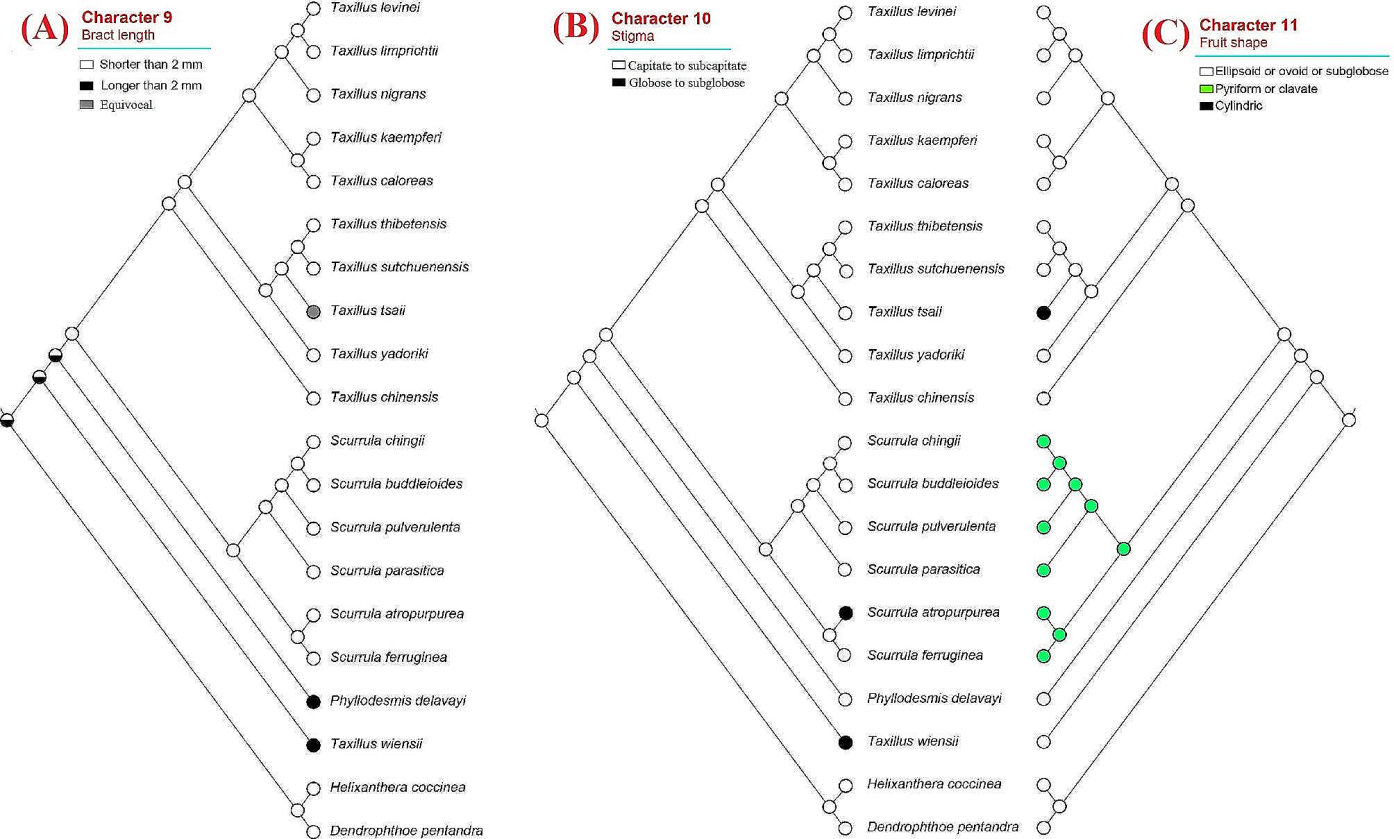
Character optimization of three morphological characters using the molecular phylogeny. **(A)** Bract length; **(B)** stigma; **(C)** fruit shape

We explored the state evolution of eleven morphological characters in Scurrulinae using the combined phylogeny. Our results showed that “trichomes present” on young stems were the ancestral state in the subtribe, and “glabrous” was the derived state (character 1, Fig. 2A). Similarly, “trichomes present” leaves and corollas were the ancestral states, and “glabrous” was the derived state in the subtribe (characters 3 and 5, Fig. 2B, D). The ancestral state of leaf placement was “opposite or subopposite”, and “alternate” leaf was the derived state (character 2, Fig. 2C). Our analysis revealed that “ovate” was the ancestral state of bract shape and “triangular, narrow boat-shaped, and broad-triangular” were the derived character states (character 8, Fig. 3B). While the ancestral state of inflorescence type was unstable for the whole subtribe, our result indicated that inflorescence of *Taxillus*’s ancestor was umbel (character 4, Fig. 3A). Similarly, the ancestral state of corolla lobe number of the subtribe was unstable (character 6, Fig. 3C). Our results showed that ancestor of *Taxillus*, *Scurrula*, and *Phyllodesmis* had 4-merous flowers (Fig. 3C). For the character 7, “flower pedicel present” was the ancestral state, and “flower pedicel absent” was the derived state (Fig. 3D), and the latter was only seen in *Taxillus wiensii*. The ancestral state of bract length (character 9) was not resolved, however, our results showed that “bract length shorter than 2 mm” was the ancestral state of *Taxillus* and *Scurrula* (Fig. 4A). Stigma “capitate to subcapitate” was the ancestral state, while “globose to subglobose” was derived (character 10, Fig. 4B). Meanwhile, fruit shape “ellipsoid or ovoid or subglobose” was found as the ancestral state, and “pyriform or clavate” and “cylindric” were derived in the subtribe (character 11, Fig. 4C).

### Divergence times estimation

**Fig. 5:**
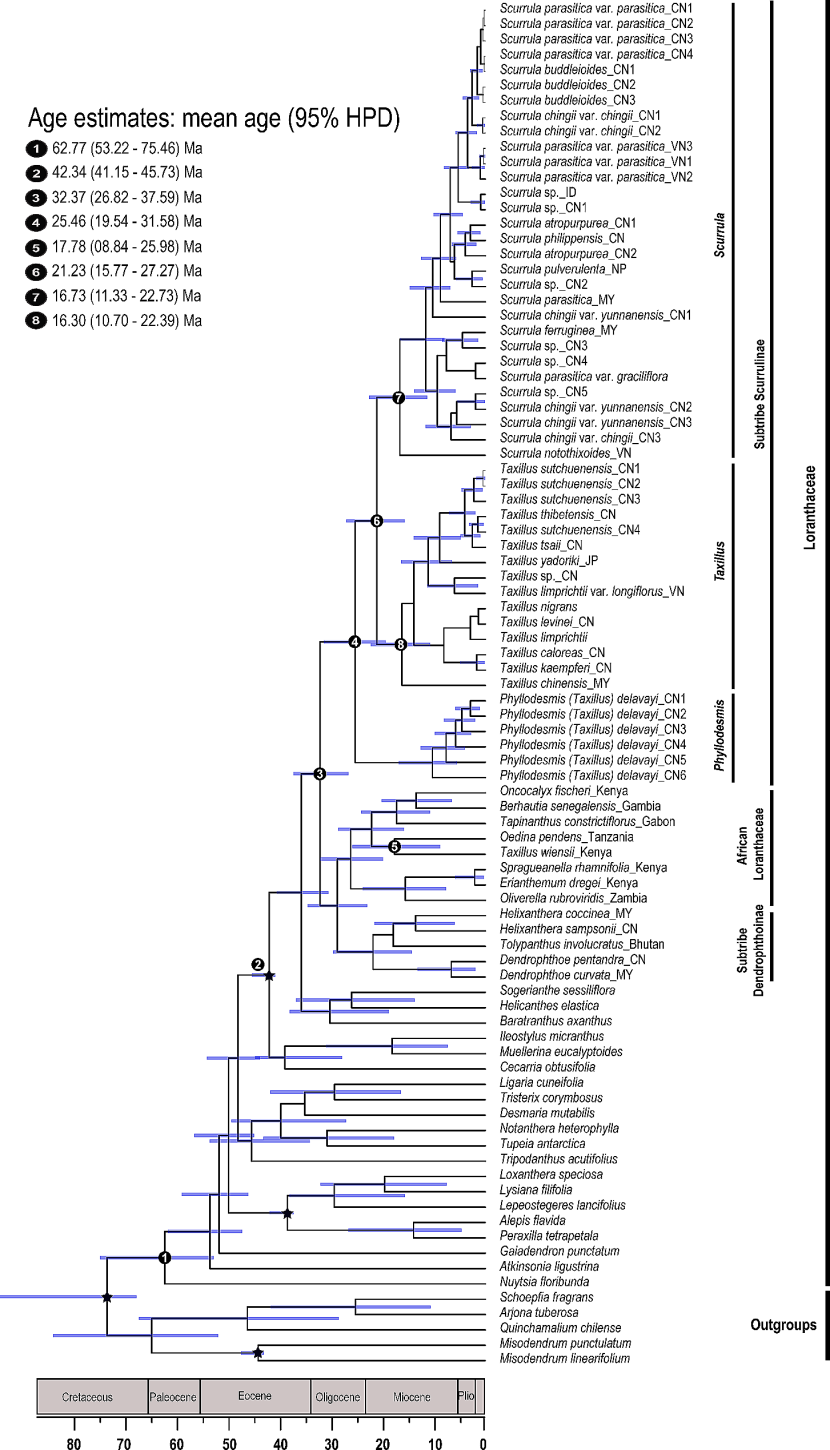
Maximum clade credibility tree inferred from BEAST based on the combined datasets of six DNA regions. The bars around node ages indicate 95% highest posterior density intervals. Node constraints are indicated with stars. Nodes of interests were marked as 1–6. Abbreviations: CN: China, VN: Vietnam, ID: Indonesia; MY: Malaysia, JP: Japan, NP: Nepal

The divergence time estimations for the Loranthaceae and subtribe Scurrulinae are shown in Fig. 5. The crown age of the family Loranthaceae was estimated to be approximately 62.77 Ma (95% HPD: 53.22–75.46 Ma; node 1, Fig. 5). The tribe Lorantheae initially diverged around 42.34 Ma (95% HPD: 41.15–45.73 Ma; node 2, Fig. 5).

The subtribe Scurrulinae split from the ancestors of the subtribe Dendrophthoinae plus African Loranthaceae at 32.37 Ma (95% HPD: 26.82–37.59 Ma; node 3, Fig. 5). Within Scurrulinae, *Phyllodesmis* diverged from the remaining taxa of the subtribe at 25.46 Ma (95% HPD: 19.54–31.58 Ma; node 4, Fig. 5). The two genera *Scurrula* and *Taxillus* split from each other at 21.23 Ma (95% HPD: 15.77–27.27 Ma; node 6, Fig. 5), then started to diversify at 16.73 Ma (95% HPD: 11.33–22.73 Ma; node 7, Figs. 5) and 16.30 Ma (95% HPD: 10.70–22.39 Ma; node 8, Fig. 5), respectively. Additionally, *Taxillus wiensii* separated with *Oedina pendens* at 17.78 Ma (95% HPD: 8.84–25.98 Ma; node 5, Fig. 5).

### Ancestral range reconstruction

**Fig. 6:**
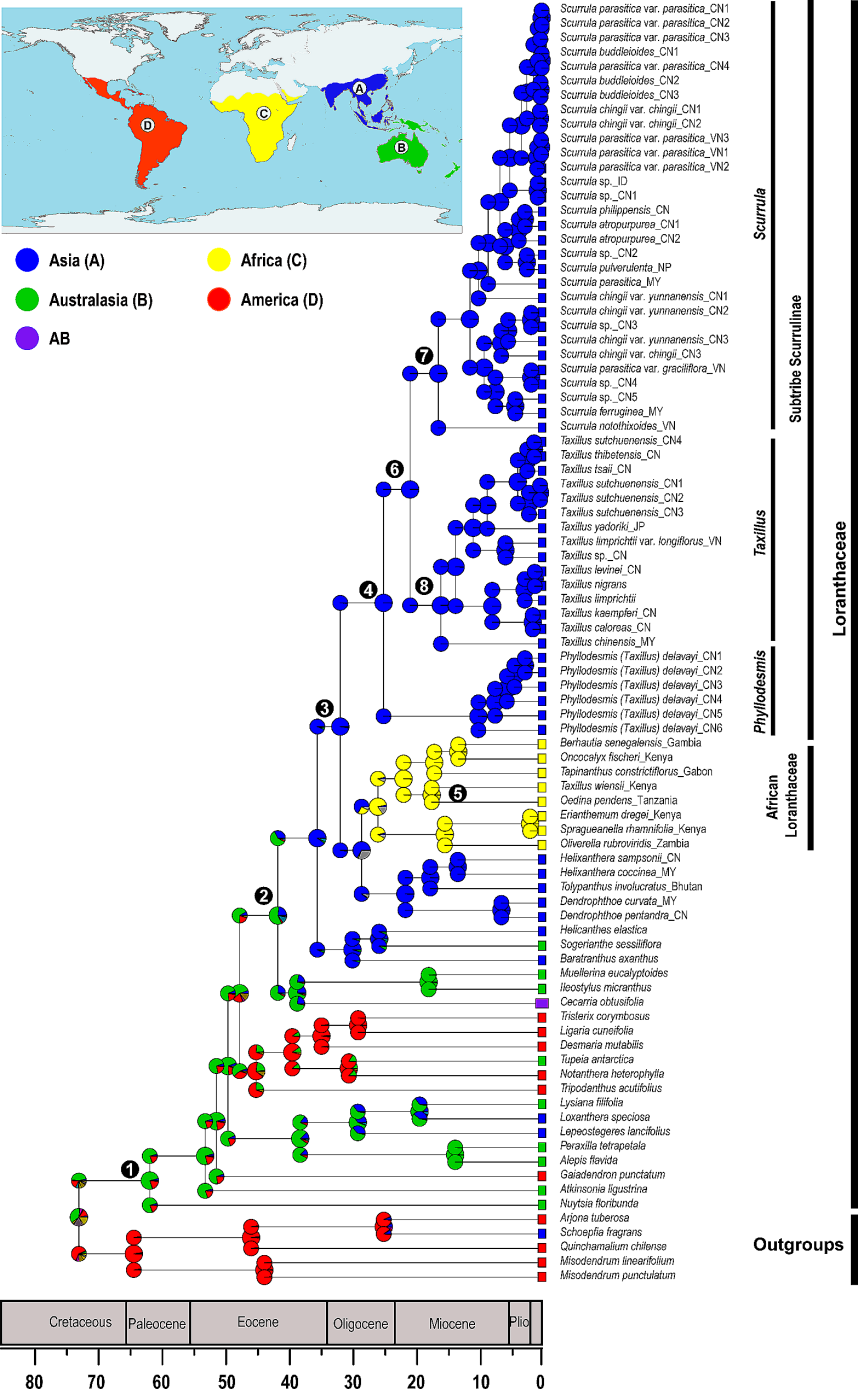
Ancestral range reconstruction of Scurrulinae by BioGeoBEARS (*j* = 0.0169, LnL = − 48.35). Geologic time scale is shown at the bottom. Numbers outside nodes correspond to the node numbers in Fig. 5. Area abbreviations are as follows: A = Asia; B = Australasia; C = Africa (including Arabian Peninsula); D = Americas. Abbreviations: CN: China, VN: Vietnam, ID: Indonesia; MY: Malaysia, JP: Japan, NP: Nepal

Results of ancestral range reconstruction analyses from BioGeoBEARS and Bayes-DIVA are congruent. Among the six models used in our BioGeoBEARS analysis, models including three parameters had higher log likelihood values than models including two parameters (Table 2), this result shows that jump speciation such as dispersal between non-adjacent areas is an important pattern in range variation of the Loranthaceae. Moreover, the results from BioGeoBEARS analyses indicated that DEC + *j* is the best-fit biogeographic model for ancestral range reconstruction of Loranthaceae. Therefore, we are only presenting the reconstruction from BioGeoBEARS using the DEC + *j* model (Fig. 6). Additionally, the result from Bayes-DIVA is exhibit in Fig. S1. Node numbers in Figs. 5 and 6 are consistent, and we summarize the divergence time estimations and ancestral range reconstruction in Table 3. Biogeographic stochastic mapping (BSM) under the DEC + *j* model indicated that most biogeographic events comprised within-area speciation (78%) and dispersals (20.7%), with very few (1.3%) vicariant events (Table S4). The stem group of Scurrulinae was estimated to originate from Asia (area A) (node 3, Fig. 6), and Scurrulinae subsequently diversified in Asia during the Oligocene. The genus *Phyllodesmis* was estimated to originate in Asia during the Late Oligocene (node 4, Fig. 6). *Taxillus* and *Scurulla* also originated in Asia and diversified during the middle Miocene (nodes 7 and 8, Fig. 6). Additionally, the only African member of Scurullinae (*Taxillus wiensii*) was estimated to diverge from its African relative during the Miocene (nodes 5, Fig. 6).

**Table 2:** Comparison of the fit of different models of biogeographic range evolution and model specific estimates for different parameters (*d* = dispersal, *e* = extinction, *j* = weight of jump dispersal (founder speciation))

Model	Parameter No	LnL	d	e	j	AIC	AIC weight
DEC	2	−48.79	4.5 × 10^− 4^	0.3161	0	102.70	0.0398
DEC + *j*	3	−48.35	2.4 × 10^− 4^	0.3255	0.0169	96.33	0.960
DIVALIKE	2	−49.60	4.0 × 10^− 4^	0.1499	0	105.90	0.013
DIVALIKE + *j*	3	−51.96	3.3 × 10^− 5^	1.0 × 10^− 12^	0.0186	97.20	0.99
BAYAREALIKE	2	−62.40	5.5 × 10^− 5^	0.0168	0	115.45	1
BAYAREALIKE + *j*	3	−49.12	1.2 × 10^− 4^	0.0058	0.0215	109.23	0.65

**Table 3:** Summary of supported clades, divergence age estimation (Ma) by BEAST and ancestral range reconstructions by Bayes-DIVA and maximum likelihood. Node numbers refer to Figs. 5 and 6. Area abbreviations are as follows: A = Asia; B = Australasia; C = Africa (including Arabian Peninsula); D = Americas

Node	Bayesian PP	Age estimates mean (95% HPD) [Ma]	BioGeoBEARS (DEC + j)	Likelihood-DEC (relative probability)	Bayes-DIVA (maximum probability)
**1**	1.0	59.38 (52.58, 65.59)	A	A|A (0.94)	A (94.64)
**2**	1.0	51.30 (45.71, 57.42)	C	C|C (0.95)	C (97.22)
**3**	1.0	50.04 (44.61, 55.90)	A	A|A (0.94)	A (86.93)
**4**	1.0	48.05 (42.39, 53.15)	A	A|A (0.94)	A (75.52)
**5**	< 0.90	39.33 (36.48, 42.12)	A	A|A (0.94)	A (65)

## Discussion

### Phylogenetic relationship

With extensive taxon and gene sampling, this study retrieves a well-supported topology for most clades, although some subclades within the genera *Taxillus* and *Scurrula* exhibit weak support (Fig. 1). Our results demonstrate that the subtribe Scurrulinae is non-monophyletic, with one species, *Taxillus wiensii*, nested within the African Loranthaceae clade. *Phyllodesmis* is strongly supported as sister to the two genera, *Taxillus* and *Scurrula*. Morphological characters are useful to support relationships within the subtribe, particularly the glabrous young stems and leaves can distinguish *Phyllodesmis* from *Taxillus* and *Scurrula* (Fig. 2A, B). *Taxillus wiensii* clade is characterized by bract shape, corolla lobe number, flower pedicel (Fig. 3B, C, D), while, the clade of *Taxillus* and *Scurrula* is characterized by bract length (Fig. 4A). Our character optimizations suggest that the two genera *Taxillus* and *Scurrula* are very similar in morphology, and they share ancestral morphological states of most characters. However, they have evolved differently in the shape of fruit and stigma (Fig. 4B, C). Based on our observation of specimens and fresh plants in the field, fruits of *Scurrula* are always pyriform or clavate sometimes, while those of *Taxillus* are usually ellipsoid, ovoid, or cylindrical.

*Scurrula* comprises around 50 species in China and Southeast Asia. Our phylogenetic analysis revealed unexpected relationships between the *S. parasitica* complex and the *S. chingii* complex (Fig. 1). *S. parasitica* was found to be a complex member in this study based on molecular data. There are two varieties of *S. parasitica*: *S. parasitica* var. *parasitica* and *S. parasitica* var. *graciliflora*. Our results demonstrated that the different individuals of *S. parasitica* var. *parasitica* and *S. parasitica* var. *graciliflora* did not form a monophyletic clade (Fig. 1). Moreover, *S. parasitica* var. *graciliflora* can be easily distinguished from *S. parasitica* var. *parasitica* by its greenish-yellow corolla (versus red corolla in *S. parasitica* var. *parasitica*). Based on our findings, we suggest that *S. parasitica* var. *graciliflora* should be redefined as a separate species. A detailed treatment of this taxon will be provided in a future study.

*Scurrula chingii* is composed of two varieties: *S. chingii* var. *yunnanensis* and *S. chingii* var. *chingii* [8]. Both varieties are non-monophyletic by Liu et al. [5] and our study (Fig. 1). Although the position of *S. chingii* var. *yunnanensis* is uncertain in our phylogenetic tree, it is distant from *S. chingii* var. *chingii* as reported by Liu et al. [5]. Furthermore, *S. chingii* var. *yunnanensis* can be easily distinguished from *S. chingii* var. *chingii* based on several characteristics, including glabrous leaf blade surfaces (versus rusty red tomentose or glabrous abaxial surface in *S. chingii* var. *chingii*), shorter peduncle and floral axis less than 10 mm (vs. 10–25 mm in *S. chingii* var. *chingii*), and lanceolate corolla lobes (versus subspatulate lobes in *S. chingii* var. *chingii*). Additionally, *Scurrula chingii* var. *yunnanensis* is endemic to Yunnan (China), while *S. chingii* var. *chingii* is distributed in Guangxi, southern Yunnan (China), and northern Vietnam. Based on our results, *S. chingii* var. *yunnanensis* should be redefined to the species rank. The detail treatment will be provided in a future study.

*Taxillus* includes approximately 35 species from tropical Asia (India and Sri Lanka to China, Japan, Philippines, Borneo) and Africa (Kenya coast). *Taxillus* is generally characterized by low host specificity. *Taxillus chinensis* (DC.) Danser, a Malesian species, is widely distributed in west of Charles’s Line. On the other hand, *T. wiensii* Polhill, the only species of *Taxillus* in East Africa, has a narrow distribution limited to the Kenya coast.

Polhill and Wiens [7] suggested that although the morphology of *T. wiensii* is similar to the species of *Taxillus* in Sri Lanka, the flowers of *T. wiensii* appear different from the Asiatic species due to the erect and possibly spontaneously open corolla-lobes. Additionally, Polhill and Wiens [7] proposed that *T. wiensii* is more comparable to the African genera that have been segregated from sections of *Taxillus* based on flower characteristics, such as sect. *Bakerella* (Tieghem) Balle, sect. *Remoti*, and sect. *Septulina*. Furthermore, all species of Loranthaceae in continental Africa and Madagascar, except *Socratina* and one species of *Septulina*, can be distinguished by their flowers that open spontaneously with erect or spreading corolla-lobes, rather than explosively as in *Taxillus*. *Bakerella* is entirely glabrous, while *Socratina* has trichomes, with a unique occurrence of fine indumentum on the inner face of the corolla-lobes. In terms of morphology, *T. wiensii* can be distinguished from the Asian *Taxillus* by its 5-merous (rather than 4 merous) flowers and bract shape (as shown in Fig. 3C). Our analyses of character optimizations indicate that *T. wiensii* and the Asian *Taxillus* have evolved differently in terms of flower and bract structure. It is difficult to improve the generic classification without detailed consideration of relationship between *T. wiensii* and the Asian *Taxillus* species [7]. Our molecular results indicate that *T. wiensii* is placed within African Loranthaceae and is clearly different from the Asian *Taxillus* (Fig. 1). Therefore, we suggest that the African *Taxillus* should be recognized as a new genus, and the detail description will be provided in a future study.

Furthermore, our molecular analyses revealed that *Taxillus limprichtii* and its variety *T. limprichtii* var. *longiflorus* (Lecomte) H. S. Kiu do not form a monophyletic group. Therefore, we recommend that their taxonomic classification be re-evaluated in future studies.

**Fig. 7:**
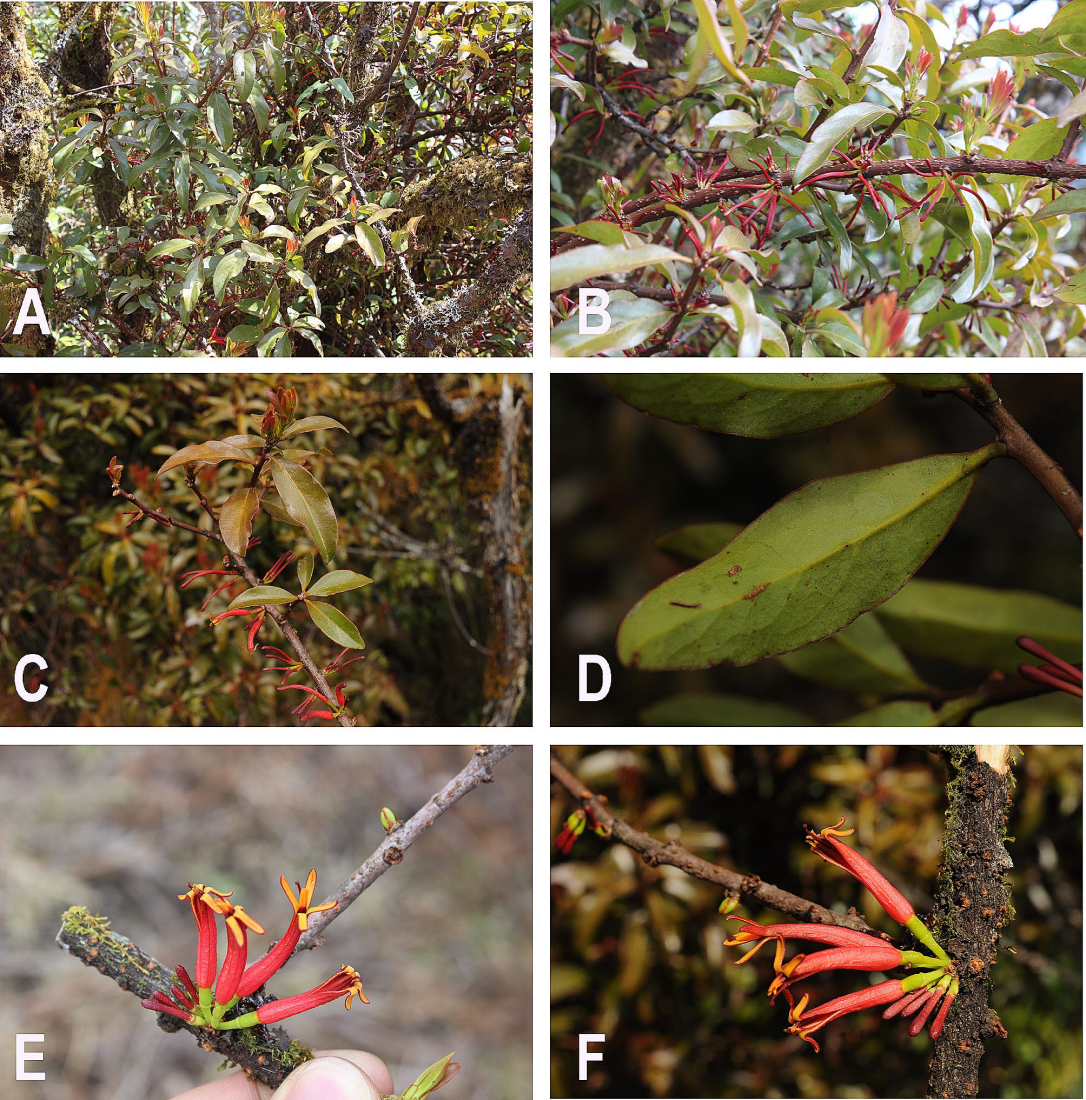
The morphological characters of *Phyllodesmis delavayi*. **A** – **B**: habits; **C**: adaxial leaf; **D**: abaxial leaf; **E** – **F**: inflorescence and flower. Photo credits: C. T. Le

*Phyllodesmis*, comprised four species, was initially described by Tieghem [11]. However, subsequent research reduced this genus to a synonym of *Taxillus*, incorporating *T. delavayi* (Tieghem) Danser, *T. kaempferi* (Candolle) Danser, *T. caloreas* (Diels) Danser, and *T. renii* H.S. Kiu [8]. Our results support *Phyllodesmis* as a distinct clade from *Taxillus* and *Scurrula* with strong support (Fig. 1). Moreover, the *Phyllodesmis* clade includes only *P. delavayi*, a species that does not parasitize species of Pinaceae, unlike the remaining three species. Furthermore, *Phyllodesmis* can be easily distinguished from all other *Taxillus* members based on characteristics of leaves alternate (as opposed to opposite or subopposite), glabrous young branchlets (trichomes not present) and both surfaces of leaves (Fig. 7). Thus, we suggest reinstating *Phyllodesmis* as a recognized genus, comprising only one species, *P. delavayi*.

### Taxonomic treatment of *Phyllodesmis*

***Phyllodesmis*** Tiegh. in Bull. Soc. Bot. France 42: 255. 1895 (Fig. 7).

Aerial parasite, small shrubs, glabrous. Leaves alternate, sometimes subopposite, pinnately veined. Inflorescences at leafless node, umbels 2–4-flowered; 1 bract subtending each flower, usually scale-like. Flowers bisexual, 4-merous, zygomorphic. Calyx ellipsoid or ovoid, rarely subglobose, base rounded, limb annular, entire or denticulate, persistent. Mature flower bud tubular, tip ellipsoid. Corolla sympetalous, slightly curved, basal portion ± inflated, split along 1 side at anthesis, lobes all reflexed toward the side away from the split, red, glabrous. Stamens inserted at base of corolla lobes; filaments short; anthers 4-loculed. Pollen grain trilobate or semilobate in polar view. Ovary 1-loculed; placentation basal. Style filiform, 4-angled; stigma usually capitate. Berry ellipsoid or ovoid, exocarp leathery, verrucose or granular, rarely smooth, pubescent or glabrous, base rounded.

*Phyllodesmis delavayi* Tieghem, Bull. Soc. Bot. France 42: 255. 1895. *Taxillus delavayi* (Tieghem) Danser, Verh. Kon. Ned. Akad. Wetensch., Afd. Natuurk., 29(6): 123. 1933. Type: China, Yunnan, Dali, Feb. 1887, *Delavay 2620* (P, syntypes barcodes P00756268!, P00756269!, P00756270!, P00756271!)

#### Morphology

—Aerial parasite, small shrubs 0.5–1 m tall, glabrous. Branches grayish, brown to almost blackish, usually lenticellate, glabrous when young, later often longitudinally fissured. Leaves simple, alternate, sometimes sub-opposite; petiole sub-sessile, 3–5 mm long; leaf blade elliptic to lanceolate, 3–6 × 1.3–2.5 cm, leathery, glabrous on both surfaces, lateral veins 3–4 pairs, adaxially somewhat obscure, base cuneate, slightly decurrent, apex obtuse, margin entire, or slightly undulate, brown, young leaves brown to reddish, adaxially glossy, green, abaxially pale green. Inflorescence with 1–2 umbels, spreading along leafless branches, rarely at leaf base, 2-7-flowered; peduncle almost sessile, ca. 1–2 mm, bracteates; bracts ovate, ca. 2 mm long, glabrous. Pedicel terete, 5–15 mm long, pale green, glabrous. Calyx ellipsoid, ca. 2.5 mm long, limb annular, entire or minutely 4-toothed. Mature bud 4–5 cm, tip ellipsoid, tip inside yellow. Corolla red, slightly curved, glabrous, lobes linear-lanceolate, 6–9 mm long, reflexed, abaxially yellow. Filaments red, short ca. 2.5 mm long; anthers orange, ca. 1.5 mm long. Ovary 1-loculed. Style red, ca. 3–5 cm long. Stigma red, capitates. Berry yellow or orange, ellipsoid, 8–10 × 3–4 mm, glabrous.

#### Phenology

—Flowering in Feb–May, fruiting in Apr–Sep.

#### Habitat

—Forests, mountain slopes; 1500–3000 m.

#### Conservation

—While not threatened as a species, and not listed under IUCN criteria, some populations of *Phyllodesmis* do require protection from over collection for medicinal use.

#### Distribution

—China: Yunnan, Guangxi, Sichuan, Xizang; Myanmar, and Vietnam: Lao Cai, Lai Chau.

#### Selected specimens examined

—VIETNAM: Lao Cai: Sapa district, San Sa Ho commune, Cat Cat village, October 2019, Van Du Nguyen, Hung Manh Nguyen, Xuan Thanh Trinh and Chi Toan Le DMTT38, DMTT39 (HN). CHINA: Sichuan: Dêrong County, Zigen, Jul 1981, *Qinghai-Tibetan Expedition Team 1734* (PE); Shimian County, 1955, *C.J. Xie 39,892* (PE). Xizang: Zayü County, Shangchayu, Jul 1980, *C.C. Ni* et al. *740* (PE). Yunnan: Wenshan County, Mt. Laojun, April 1993, *Y.M. Shui 1904* (PE); Dêqên County, Benzilan, July 1981, *Qinghai-Tibetan Expedition Team 1864* (PE).

### Historical biogeography of Scurrulinae

#### Asian origin of Scurrulinae

Our divergence time estimations for Scurrulinae are consistent with those from Grímsson et al. [26] and Liu et al. [5], and the stem age of Loranthaceae in our study is close to the results of Magallón et al. [45] and Liu et al. [5] (Table S5). The biogeographic analyses and divergence time estimations suggest that the stem group of Scurrulinae originated in Asia ca. 32.37 Ma during the Oligocene (node 3, Figs. 5 and 6; Table 3), with a crown age dating back to 25.46 Ma (95% HPD: 19.54–31.58 Ma; node 4, Figs. 5 and 6; Table 3). The Indian subcontinent began drifting from Australia-Antarctica ca. 136 Ma [44], and connected to mainland Asia ca. 44 Ma. Thus, the connection between India and Asia occurred much earlier than the origin of the Scurrulinae. According to Li et al. [46], the uplift of high mountains in Asia during the Oligocene-Miocene, combined with the southwest monsoon in Asia, probably provided ideal conditions for colonization and wide distribution of Scurrulinae. Additionally, short-distance dispersal in Asia is also important for domination or wide distribution of plants. Xiang et al. [47] suggested that the presence of tropical forests with key plant families such as Fabaceae, Fagaceae, and Rubiaceae is an important factor supporting the origin and divergence of the epiphytic plant genus *Dendrobium* (Orchidaceae) in Asia, this is consistent with the situations of aerial parasitic plants Scurrulinae or understory vegetation such as *Alpinia* (Zingiberaceae) [48] and Menispermaceae [49]. Therefore, Asian Scurrulinae, including Indian Scurrulinae, likely originated in Asia and may have spread throughout the area by birds or small animals [50–52].

The Asian Loranthaceae migrated from Australia in the late Eocene (Fig. 6). Notably, despite several species of Scurrulinae being found in Malaysia, Indonesia, and the Philippines [4, 8, 9], which are geographically close to Australasia, there is no evidence of Scurrulinae dispersing from Asia to Australasia. While several Loranthaceae genera are common to both regions, including *Lepeostegeres*, *Amylotheca*, *Decaisnina*, *Macrosolen*, and *Cecarria* [2, 7], and our study suggests that all migration events from Asia to Australasia occurred before about 35 Ma, which is earlier than the origin of Scurrulinae. Thus, the *Phyllodesmis*, *Taxillus* and *Scurrula* of Scurrulinae may be endemic genera to Asia.

Liu et al. [5] proposed that within-area speciation events are more prevalent in most of the large clades that are endemic to single areas of Loranthaceae. They suggested that dispersal without “range contractions” was the main driver of range evolution, occurring more frequently than vicariance events. Our BSM results (Table S4) are support that within-area speciation events are the main factor in creating the Asian endemic group of Scurrulinae. Dispersal events have been considered as the most common factor for worldwide distributed plants, including Loranthaceae and Scurrulinae lineages [40]. Our BSM indicates that the dispersal events of Scurrulinae occurred without “range contractions” or “founder events”, which is consistent with biogeographic history of the subtribe Scurrulinae (Table S4). After originating in Asia, Scurrulinae did not disperse to other regions. African Loranthaceae evolved from Asian ancestor and *Taxillus wiensii* originated in Africa from the African Loranthaceae ancestor. Short-distance dispersal events between proximal regions appear to have been frequent in the historical biogeography of Scurrulinae, and it may have been facilitated by the colonization or domination of Scurrulinae host plants in tropical forests.

Species of Loranthaceae are distributed worldwide [2, 4, 5, 8]. However, due to geographic distance and climate change, they are becoming endemic groups for each continent, resulting in fewer shared genera [5]. Our results suggest that Scurrulinae originated and diverged in Asia during a period when rainforests dominated the continent [48, 53] (Fig. 6). The members of this subtribe evolved and adapted to the living conditions in Asia, and the rapid climate changes, cooling, drying, and the progressive uplift of the high mountains in central Asia, especially during the late Pliocene and Pleistocene, might have promoted the diversification of Scurrulinae and prevented their dispersal to other continents [54]. Our study does not recognize any migration of Scurrulinae to other continents since the early Oligocene, except for one species of *Taxillus* that originated in Africa.

#### African origin and diversification of Taxillus Wiensii

The present study supports the placement of *Taxillus wiensii* within Africa Loranthaceae and its close relationship to *Tapinanthus constrictiflorus* (Figs. 1 and 6). Biogeographic analyses indicate that *Taxillus wiensii* originated and diversified in Africa (Fig. 6), and this species is likely not a part of Scurrulinae. *Taxillus wiensii* was considered as the only member of *Taxillus* in Africa, with dispersal from Asia to Africa proposed to explain its historical biogeography [3, 7]. However, this study confirms that the ancestor of *Taxillus wiensii* is African Loranthaceae, and this species likely evolved separately from *Taxillus* in Asia ca. 17 Ma (Figs. 5 and 6). A similar situation was encountered in the genus *Helixanthera*, with Liu et al. [5] demonstrating differences between African and Asian *Helixanthera* and suggesting that African *Helixanthera* may be recognized as a distinct genus in the future studies.

## Conclusion

Based on comprehensive taxon sampling of the subtribe Scurrulinae, this study strongly supports the relationship among genera of the subtribe. *Phyllodesmis* is recognized as a separate genus from its allies *Taxillus* and *Scurrula* while African *Taxillus* may be treated as a new genus from Africa in the future studies. The mistletoe Scurrulinae originated in Asia during the Oligocene, and then was widespread in Asia and did not disperse to other areas. *Taxillus* and *Scurrula* diverged during the climatic optimum in the middle Miocene. African *Taxillus* originated in Africa from African Loranthaceae approximately 17 Ma, clearly different from *Taxillus* in Asia. Diversification of Scurrulinae and the development of endemic species in Asia may have been promoted by the fast-changing climate, including cooling, drying, and the progressive uplift of the high mountains in central Asia, especially during the late Pliocene and Pleistocene.

### Electronic supplementary material

Below is the link to the electronic supplementary material.

Supplementary Material 1

## Data Availability

All the sequences generated in this study are deposited in the NCBI database (https://www.ncbi.nlm.nih.gov/, accessed on 19 December 2023), under the following accession number: OR964417-OR964480, OR976247-OR976261, OR987685-OR987719, and PP002640-PP002689. Other data used in this study can be found at the Science Data Bank (10.57760/sciencedb.18240).
