# Supplementary material for: Phylogeny, character evolution and historical biogeography of Scurrulinae (Loranthaceae): new insights into the circumscription of the genus Taxillus
Source: BMC Plant Biol. 2024 May 22;24:440. doi: 10.1186/s12870-024-05126-0 (PMC11110394; doi:10.1186/s12870-024-05126-0)
Supplement: Supplementary file 1 — Supplementary Material 1 [file 12870_2024_5126_MOESM1_ESM.doc]

**Table S1.** Voucher information and GenBank accession numbers for DNA sequences generated or used in this study. The sequences generated in this study “OR9”, and “PP0”. “–” indicates missing data.

| **Species** | **Voucher/Source** | **Country of origin** | **Identifier** | **LSU** | **SSU** | ***mat*K** | ***rbc*L** | ***trn*L-F** | **ITS** |
| --- | --- | --- | --- | --- | --- | --- | --- | --- | --- |
| *Alepis flavida* (Hook.f.) Tiegh. | *B. Molloy s.n.* (SIU) | New Zealand | D. L. Nickrent | EF464474 | L24139 | EF464508 | KT626664 | EF464481 | DQ333847 |
| *Arjona tuberosa* Cav. | *V. Melzheimer s.n.* (SIU) | Argentina | D. L. Nickrent | EF464480 | EF464468 | EF464513 | EF464532 | EF464483 | MH660622 |
| *Atkinsonia ligustrina* (Lindl.) F.Muell. | *D. Watson 4458* (SIU) | Australia | D. L. Nickrent | EF464475 | EF464464 | DQ787444 | EF464526 | DQ788714 | DQ333865 |
| *Baratranthus axanthus* (Korth.) Miq. | *D. L. Nickrent 4029* (SIU) | Malaysia | D. L. Nickrent | EU544357 | EU544317 | EU544414 | – | EU544478 | DQ333838 |
| *Berhautia senegalensis* Balle | *M. Jones s.n.* (SIU) | Gambia | D. L. Nickrent | EU544360 | EU544320 | EU544417 | – | – | – |
| *Cecarria obtusifolia* (Merr.) Barlow | *B. Hyland 16493* (QRS) | Australia | D. L. Nickrent | EU544361 | EU544321 | EU544418 | EU544467 | EU544481 | DQ333836 |
| *Dendrophthoe pentandra* (Linn.) Miq. | *Z. D. Chen GX058* (PE) | China | B. Liu & C. T. Le | MG999387 | MG999463 | MG999412 | MG999432 | MG999484 | – |
| *Dendrophthoe curvata* (Blume) Miq. | *D. L. Nickrent 4012* (SIU) | Malaysia | D. L. Nickrent | EU544367 | EU544325 | EU544424 | – | – | – |
| *Desmaria mutabilis* (Poepp. & Endl.) Tiegh. Ex T.Durand & B.D.Jacks. | *G. Amico s.n.* (SIU) | Chile | D. L. Nickrent | EF464476 | EF464465 | EF464509 | EF464527 | EF464486 | DQ333852 |
| *Erianthemum dregei* (Eckl. & Zeyh.) Tiegh. | *S. A. Robertson s.n.* (SIU) | Kenya | D. L. Nickrent | EU544371 | L25679 | EU544428 | – | EU544488 | DQ333831 |
| *Gaiadendron punctatum* (Ruiz & Pav.) G. Don | *S. Sargent s.n.* (SIU) | Costa Rica | D. L. Nickrent | DQ790209 | L24143 | DQ787445 | L26072 | DQ340617 | DQ333866 |
| *Helicanthes elastica* (Desv.) Danser | *Pradeep 5342* (SIU) | India | D. L. Nickrent | EU544375 | EU544328 | EU544432 | – | – | – |
| *Helixanthera coccinea* (Jack) Danser | *D. L. Nickrent 4019* (SIU) | Malaysia | D. L. Nickrent | EU544373 | – | EU544430 | – | EU544490 | OR976247 |
| *Helixanthera sampsonii* (Hance) Danser | *s.n. SCBGP388* | China | J. Liu | – | – | KP093921 | KP094864 | – | – |
| *Ileostylus micranthus* Tiegh. | *B. Molloy s.n.* (SIU) | New Zealand | D. L. Nickrent | EU544376 | EU544329 | EU544433 | EU544471 | EU544491 | DQ333841 |
| *Lepeostegeres lanceifolius* Danser | *Calvin et al. B27* (SAR) | Malaysia | D. L. Nickrent | EU544379 | – | EU544435 | – | – | – |
| *Ligaria cuneifolia* (Ruiz & Pav.) Tiegh. | *G. Amico s.n.* (SIU) | Chile | D. L. Nickrent | EF464477 | L24152 | EF464510 | EF464528 | DQ442940 | DQ333853 |
| *Loxanthera speciosa* Blume | *D. L. Nickrent 4026* (SIU) | Malaysia | D. L. Nickrent | EU544382 | EU544332 | EU544437 | – | EU544495 | – |
| *Lysiana filifolia* Barlow | *D. L. Nickrent 4449* (SIU) | Australia | D. L. Nickrent | EU544383 | EU544333 | EU544438 | – | EU544496 | – |
| *Misodendrum linearifolium* DC. | *G. Amico 136* (BCRU) | Chile | D. L. Nickrent, | DQ790211 | L24397 | DQ787438 | L26074 | DQ788712 | – |
| *Misodendrum punctulatum* Banks ex DC. | *R. Vidal–Russell 61, 62* (BCRU) | Argentina | D. L. Nickrent | KP263250 | KP263284 | DQ787443 | EF464531 | DQ788711 | – |
| *Muellerina eucalyptoides* (DC.) Barlow | *D. Watson s.n.* (SIU) | Australia | D. L. Nickrent | EU544385 | EU544335 | EU544440 | EU544472 | EU544498 | – |
| *Notanthera heterophylla* (Ruiz & Pav.) G. Don | *C. Aeodo 7202* (MA) | Chile | D. L. Nickrent | EF464478 | EF464466 | EF464511 | EF464529 | DQ442939 | DQ333855 |
| *Nuytsia floribunda* R.Br. | *B. Lamont s.n.* (SIU) | Australia | D. L. Nickrent | DQ790210 | DQ790103 | DQ787446 | DQ790134 | DQ788716 | DQ333867 |
| *Oedina pendens* (Engl. & K. Krause) Polhill & Wiens | *R. E. Gereau & C. J. Kayombo 4213* (MO) | Tanzania | D. L. Nickrent | EU544386 | – | EU544441 | – | EU544499 | – |
| *Oliverella rubroviridis* Tiegh. | *N. B. Zimba et al. 1097* (MO) | Zambia | D. L. Nickrent | EU544387 | EU544337 | EU544442 | – | – | – |
| *Oncocalyx fischeri* (Engl.) M.G. Gilbert | *Z. D. Chen et al. 28942* (PE) | Kenya | B. Liu & C. T. Le | MG999394 | MG999469 | MG999417 | MG999441 | MG999492 | OR976248 |
| *Peraxilla tetrapetala* (L. F.) Tiegh. | *B. Molloy s.n.* (SIU) | New Zealand | D. L. Nickrent | EU544390 | EU544340 | EU544446 | JQ933439 | EU544502 | DQ333846 |
| *Phylodesmis delavayi* Tiegh. | *B. Liu et al. 2199* (PE) | China | B. Liu & C. T. Le | OR964417 | OR964449 | PP002640 | PP002655 | OR987685 | OR976249 |
| *Phylodesmis delavayi* Tiegh. | *Z. D. Chen 654* (PE) | China | B. Liu & C. T. Le | OR964418 | OR964450 | PP002641 | PP002656 | OR987686 | OR976250 |
| *Phylodesmis delavayi* Tiegh. | *B. Liu et al. 26245* (PE) | China | B. Liu & C. T. Le | OR964419 | OR964451 | – | PP002657 | OR987687 | MH117790 |
| *Phylodesmis delavayi* Tiegh. | *H. S He et al. 89-2153* (PE) | China | B. Liu & C. T. Le | OR964420 | OR964452 | – | PP002658 | OR987688 | – |
| *Phylodesmis delavayi* Tiegh. | *Z. D. Chen 90* (PE) | China | B. Liu & C. T. Le | OR964421 | OR964453 | PP002642 | PP002659 | OR987689 | OR976251 |
| *Phylodesmis delavayi* Tiegh. | *L. M. Lu 2008026* (PE) | China | B. Liu & C. T. Le | OR964422 | OR964454 | PP002643 | PP002660 | OR987690 | OR976252 |
| *Quinchamalium chilense* Molina | *R. Vidal-Russell s.n.; J.R.I. Wood 9149* (SIU; K) | Argentina; Bolivia | D. L. Nickrent; H. J. Su | KP263257 | EF464469 | EF464514 | EF464533 | EF464491 | – |
| *Schoepfia fragrans* Wall. | *B. Liu 2500* (PE) | China | B. Liu & C. T. Le | MG999400 | MG999474 | MG999422 | MG999449 | MG999498 | OR976253 |
| *Scurrula atropurpurea* (Blume) Danser | *B. Liu et al. 27862* (PE) | China | B. Liu & C. T. Le | OR964423 | OR964455 | PP002644 | PP002661 | OR987691 | – |
| *Scurrula atropurpurea* (Blume) Danser | *J. X. Su SJX178* (PE) | China | B. Liu & C. T. Le | OR964424 | OR964456 | PP002645 | PP002662 | OR987692 | – |
| *Scurrula buddleioides* (Desr.) G. Don | *Z. J. Qiu 0096* (PE) | China | B. Liu & C. T. Le | MG999402 | MG999475 | MG999423 | MG999451 | MG999499 | OR976254 |
| *Scurrula buddleioides* (Desr.) G. Don | *B. Liu et al. 27764* (PE) | China | B. Liu & C. T. Le | OR964425 | OR964457 | – | PP002663 | OR987693 | OR976255 |
| *Scurrula buddleioides* (Desr.) G. Don | *B. Liu et al. 27820* (PE) | China | B. Liu & C. T. Le | OR964426 | OR964458 | PP002646 | PP002664 | OR987694 | – |
| *Scurrula chingii* var. *yunnanensis* H. S. Kiu in C. Y. Wu & H. W. Li | *B. Liu 2516* (PE) | China | B. Liu & C. T. Le | MG999404 | MG999477 | MG999425 | MG999453 | MG999501 | – |
| *Scurrula chingii* var. *yunnanensis* H. S. Kiu in C. Y. Wu & H. W. Li | *B. Liu et al. 5907* (PE) | China | B. Liu & C. T. Le | OR964427 | OR964459 | – | PP002665 | OR987695 | – |
| *Scurrula chingii* var. *yunnanensis* H. S. Kiu in C. Y. Wu & H. W. Li | *B. Liu et al. 5985* (PE) | China | B. Liu & C. T. Le | OR964428 | OR964460 | – | PP002666 | OR987696 | – |
| *Scurrula chingii* (W.C. Cheng) H.S. Kiu | *B. Liu 1736* (PE) | China | B. Liu & C. T. Le | MG999403 | MG999476 | MG999424 | MG999452 | MG999500 | OR976256 |
| *Scurrula chingii* var. *chingii* | *B. Liu et al. 27484* (PE) | China | B. Liu & C. T. Le | – | OR964461 | – | PP002667 | OR987697 | – |
| *Scurrula chingii* var. *chingii* | *YN-ET 043* (PE) | China | B. Liu & C. T. Le | OR964429 | OR964462 | PP002647 | PP002668 | OR987698 | – |
| *Scurrula ferruginea* (Jack) Danser | *D. L. Nickrent 4008* (SIU) | Malaysia | D. L. Nickrent | EU544395 | EU544343 | EU544451 | KF114863 | EU544505 | DQ333827 |
| *Scurrula notothixoides* (Hance) Danser | *Z. D. Chen VN0617* (PE) | Vietnam | B. Liu & C. T. Le | OR964430 | – | – | PP002669 | OR987699 | – |
| *Scurrula parasitica* L. | *D. L. Nickrent 4004* (SIU); *T. Yang BZXHDGK0047* (PE) | Malaysia; China | D. L. Nickrent; B. Liu & C. T. Le | EU544397 | EU544345 | EU544451 | MG999454 | MG999502 | OR976257 |
| *Scurrula parasitica* var. *parasitica* | *Z. D. Chen GX096* (PE) | China | B. Liu & C. T. Le | OR964431 | – | – | – | OR987700 | – |
| *Scurrula parasitica* var. *parasitica* | *Z. D. Chen GX098* (PE) | China | B. Liu & C. T. Le | OR964432 | OR964463 | – | PP002670 | OR987701 | – |
| *Scurrula parasitica* var. *parasitica* | *Z. D. Chen GX102* (PE) | China | B. Liu & C. T. Le | OR964433 | OR964464 | – | PP002671 | OR987702 | – |
| *Scurrula parasitica* var. *parasitica* | *Z. D. Chen VN0026* (PE) | Vietnam | B. Liu & C. T. Le | OR964434 | OR964465 | – | PP002672 | OR987703 | – |
| *Scurrula parasitica* var. *parasitica* | *Z. D. Chen VN0040* (PE) | Vietnam | B. Liu & C. T. Le | OR964435 | OR964466 | – | PP002673 | OR987704 | – |
| *Scurrula parasitica* var. *parasitica* | *Z. D. Chen VN0055* (PE) | Vietnam | B. Liu & C. T. Le | OR964436 | OR964467 | – | PP002674 | OR987705 | – |
| *Scurrula parasitica* var. *parasitica* | *Z. D. Chen GX075* (PE) | China | B. Liu & C. T. Le | OR964437 | OR964468 | PP002648 | PP002675 | OR987706 | – |
| *Scurrula parasitica* var *graciliflora* | – | – | Y. H. Li | – | – | – | HQ317779 | AY191141 | – |
| *Scurrula philippensis* (Cham. & Schltdl.) G. Don | *T. Yang BZXHDGK0054* (PE) | China | B. Liu & C. T. Le | MG999405 | MG999478 | MG999426 | MG999455 | MG999503 | OR976258 |
| *Scurrula pulverulenta* (Wall.) G. Don | *M. Devkota 661* (KATH) | Nepal | D. L. Nickrent | EU544396 | EU544344 | EU544452 | – | – | – |
| *Scurrula* sp. | *B. Liu et al. 27500* (PE) | China | B. Liu & C. T. Le | – | OR964469 | – | PP002676 | OR987707 | – |
| *Scurrula* sp. | *X.H Jin & Arief 4414* (PE) | Indonesia | B. Liu & C. T. Le | – | OR964470 | – | PP002677 | – | – |
| *Scurrula* sp. | *B. Liu et al. 27821* (PE) | China | B. Liu & C. T. Le | OR964438 | OR964471 | – | PP002678 | OR987708 | – |
| *Scurrula* sp. | *B. Liu et al. 27869* (PE) | China | B. Liu & C. T. Le | OR964439 | OR964472 | PP002649 | PP002679 | OR987709 | – |
| *Scurrula* sp. | *M. Sun 53* (PE) | China | B. Liu & C. T. Le | OR964440 | OR964473 | PP002650 | PP002680 | OR987710 | – |
| *Scurrula* sp. | *Z. D. Chen 476* (PE) | Indonesia, Sulawesi | B. Liu & C. T. Le | OR964441 | OR964474 | PP002651 | PP002681 | OR987711 | – |
| *Sogerianthe sesailiflora* (Danser) Danser | *D. L. Nickrent et al. 4467* (WAU) | Papua New Guinea | D. L. Nickrent | EU544400 | EU544348 | EU544455 | – | EU544508 | – |
| *Spragueanella rhamnifolia* (Engl.) Balle | *S. A. Robertson, D. Wiens, C. Calvin 5452* (MO) | Kenya | D. L. Nickrent | EU544401 | – | EU544456 | – | – | – |
| *Tapinanthus constrictiflorus* (Engl.) Danser | *J. J. Wieringa 2860* (WAG) | Gabon | D. L. Nickrent | EU544404 | L24422 | EU544459 | EU213529 | EU544511 | – |
| *Taxillus caloreas* (Diels) Danser | *T.Naito et al. 658* (PE) | China | B. Liu & C. T. Le | OR964442 | OR964475 | PP002652 | PP002682 | OR987712 | – |
| *Taxillus chinensis* (DC.) Danser | *D. L. Nickrent 4032* (SIU); *Z. D. Chen & C. T. Le 36* (PE) | Malaysia; Vietnam | D. L. Nickrent; B. Liu & C. T. Le | EU544405 | EU544350 | EU544460 | MG999456 | MG999504 | – |
| *Taxillus kaempferi* (DC.) Danser | *M. Furuse 53130* (PE) | Japan | B. Liu & C. T. Le | – | – | – | PP002683 | OR987713 | – |
| *Taxillus levinei* (Merr.) H.S. Kiu | *s.n. (PE)* | China | B. Liu & C. T. Le | OR964443 | – | – | PP002684 | – | OR976259 |
| *Taxillus limprichtii* var. *longiflorus* (Lecomte) H.S. Kiu | *Z. D. Chen VN0184* (PE) | Vietnam | B. Liu & C. T. Le | OR964444 | OR964476 | – | PP002685 | OR987714 | – |
| *Taxillus* sp. | *B. Liu et al. 27819* (PE) | China | B. Liu & C. T. Le | OR964445 | OR964477 | PP002653 | PP002686 | OR987715 | – |
| *Taxillus sutchuenensis* (Lecomte) Danser | *Z. D. Chen 20010418* (PE) | China | B. Liu & C. T. Le | MG999406 | MG999479 | MG999427 | MG999457 | MG999505 | OR976260 |
| *Taxillus sutchuenensis* (Lecomte) Danser | *A. M. Lu 104* (PE) | China, Sichuan | B. Liu & C. T. Le | OR964446 | OR964478 | PP002654 | PP002687 | OR987716 | – |
| *Taxillus sutchuenensis* (Lecomte) Danser | *B. Liu et al. 27666* (PE) | China | B. Liu & C. T. Le | OR964447 | OR964479 | – | PP002688 | OR987717 | – |
| *Taxillus sutchuenensis* (Lecomte) Danser | *B. Liu et al. 27813* (PE) | China | B. Liu & C. T. Le | OR964448 | OR964480 | – | PP002689 | OR987718 | – |
| *Taxillus thibetensi*s (Lecomte) Danser | *B. Liu 2894* (PE) | China | B. Liu & C. T. Le | MG999407 | MG999480 | MG999428 | MG999458 | MG999506 | OR976261 |
| *Taxillus tsaii* S.T. Chiu | *Z. D. Chen 20110126* (PE) | China | B. Liu & C. T. Le | MG999408 | MG999481 | MG999429 | MG999459 | MG999507 | – |
| *Taxillus yadoriki* (Maxim.) Danser | *J.Murata et al. 15494* (PE) | Japan | B. Liu & C. T. Le | – | – | – | – | OR987719 | – |
| *Taxillus limprichtii*(Grüning) H.S. Kiu | *s.n. LS15* | – | H. Yao | – | – | – | HQ317793 | – | – |
| *Taxillus nigrans* (Hance) Danser | *s.n. LS21* | – | H. Yao | – | – | – | HQ317795 | – | – |
| *Taxillus wiensii*Balle ex Polhill | *A. Robertson 7364* (RSA) | Kenya | A. Robertson | – | – | – | – | DQ340578 | DQ333829 |
| *Tolypanthus involucratus* (Roxb.) Tiegh. | *Grierson & Long 3557* (GH) | Bhutan | D. L. Nickrent | – | – | EU544461 | – | – | – |
| *Tripodanthus acutifolius*(Ruiz & Pav.) Tiegh. | *Wasum et al. 7586* (MO) | Brazil | D. L. Nickrent | EU544406 | L24424 | EU544462 | EU544475 | EU544513 | DQ333864 |
| *Tristerix corymbosus*(L.) Kuijt | *V. Melzheimer s.n.; G. Amico s.n.* (SIU; BCRU) | Chile | D. L. Nickrent | EF464479 | EF464467 | EF464512 | EF464530 | DQ340605 | – |
| *Tupeia antarctica* (G. Forst.) Cham. & Schltdl. | *B. Molloy s.n.* (SIU) | New Zealand | D. L. Nickrent | DQ790208 | L24425 | DQ790172 | DQ790133 | EF464494 | DQ333850 |

The correspondence between the herbarium codes and their full names:

BCRU: Herbarium of National University of Comahue, Argentina.

GH: Herbarium of Harvard University, USA.

K: Herbarium of Royal Botanic Gardens, Kew, UK.

KATH: Herbarium of National Herbarium and Plant Laboratories, Nepal.

MA: Herbarium of Royal Botanic Gardens, Spain.

MO: Herbarium of Missouri Botanical Garden, USA.

PE: Herbarium of Institute of Botany, Chinese Academy of Sciences, China.

QRS: Herbarium of CSIRO, Australia.

RSA: Herbarium of California Botanic Garden, USA.

SAR: Herbarium of Department of Forestry, Malaysia.

SIU: Herbarium of Herbarium of Southern Illinois University USA.

WAG: Herbarium of Naturalis Biodiversity Center, Netherlands.

WAU: Herbarium of Wau Ecology Institute, Papua New Guinea.

**Table S2.** Gene diversity statistics for each partition, nuclear, chloroplast and the combined data sets.

|  | **No. of taxa** | **Alignment length (bp)** | **Conserv. Chars.** | **Variabl. Uninform.** | **Variabl. Inform.** |
| --- | --- | --- | --- | --- | --- |
| **LSU** | 77 | 2080 | 1582 | 288 | 210 |
| **SSU** | 75 | 1687 | 1432 | 139 | 116 |
| **ITS** | 34 | 865 | 362 | 212 | 291 |
| ***rbc*L** | 71 | 827 | 643 | 61 | 123 |
| ***mat*K** | 63 | 1416 | 562 | 319 | 535 |
| ***trn*L-F** | 76 | 903 | 474 | 217 | 212 |
| **Nuclear** | 81 | 4632 | 3376 | 639 | 617 |
| **Chloroplast** | 89 | 3146 | 1679 | 597 | 870 |
| **Combined** | 89 | 7778 | 5055 | 1236 | 1487 |

**Table S3**. Morphological matrix of the subtribe Scurrulinae. ?: missing data.

| **Species** | **01** | **02** | **03** | **04** | **05** | **06** | **07** | **08** | **09** | **10** | **11** |
| --- | --- | --- | --- | --- | --- | --- | --- | --- | --- | --- | --- |
| *Taxillus thibetensis* | 1 | 0 | 1 | 0 | 1 | 0 | 1 | 2 | 0 | 0 | 0 |
| *Taxillus chinensis* | 1 | 0 | 1 | 0 | 1 | 0 | 1 | 3 | 0 | 0 | 0 |
| *Taxillus levinei* | 1 | 0 | 1 | 0 | 1 | 0 | 1 | 3 | 0 | 0 | 0 |
| *Taxillus tsaii* | 1 | 0 | 1 | 0 | 1 | 0 | 1 | 3 | ? | 0 | 2 |
| *Taxillus nigrans* | 1 | 0 | 1 | 2 | 1 | 0 | 1 | 3 | 0 | 0 | 0 |
| *Taxillus sutchuenensis* | 1 | 0 | 1 | 1 | 1 | 0 | 1 | 2 | 0 | 0 | 0 |
| *Taxillus yadoriki* | 1 | 0 | 1 | 0 | 1 | 0 | 1 | 2 | 0 | 0 | 0 |
| *Taxillus kaempferi* | 1 | 1 | 1 | 0 | 0 | 0 | 1 | 2 | 0 | 0 | 0 |
| *Taxillus caloreas* | 1 | 1 | 1 | 0 | 0 | 0 | 1 | 2 | 0 | 0 | 0 |
| *Taxillus limprichtii* | 1 | 0 | 1 | 0 | 1 | 0 | 1 | 2 | 0 | 0 | 0 |
| *Taxillus wiensii* | 1 | 0 | 1 | ? | 1 | 1 | 0 | 1 | 1 | 1 | 0 |
| *Scurrula pulverulenta* | 1 | 0 | 1 | 1 | 1 | 0 | 1 | 2 | 0 | 0 | 1 |
| *Scurrula parasitica* | 1 | 0 | 1 | 1 | 1 | 0 | 1 | 0 | 0 | 0 | 1 |
| *Scurrula buddleioides* | 1 | 0 | 1 | 1 | 1 | 0 | 1 | 2 | 0 | 0 | 1 |
| *Scurrula chingii* | 1 | 0 | 1 | 1 | 1 | 0 | 1 | 2 | 0 | 0 | 1 |
| *Scurrula atropurpurea* | 1 | 0 | 1 | 1 | 1 | 0 | 1 | 0 | 0 | 1 | 1 |
| *Scurrula ferruginea* | 1 | 0 | 1 | 1 | 1 | 0 | 1 | 0 | 0 | 0 | 1 |
| *Phyllodesmis delavayi* | 0 | 1 | 0 | 0 | 0 | 0 | 1 | 2 | 1 | 0 | 0 |
| *Helixanthera coccinea* | 1 | 1 | 1 | 1 | 1 | 0 | 1 | 0 | 0 | 0 | 0 |
| *Dendrophthoë pentandra* | 1 | 0 | 0 | 1 | 1 | 1 | 1 | 2 | 0 | 0 | 0 |

**Table S4**. Summary of biogeographical stochastic mapping counts for the Scurrulinae using the DEC+*j* model.

| **Mode** | **Type** | **Mean (SD)** | **%** |
| --- | --- | --- | --- |
| **Within-area speciation** | Speciation | 83.95 (1.65) | 75.63 |
|  | Speciation–subset | 2.56 (1.45) | 2.3 |
| **Dispersal** | Founder events | 0 | 0 |
|  | Range expansions | 23 (3.43) | 20.73 |
|  | Range contractions | 0 | 0 |
| **Vicariance** | Vicariance | 1.49 (1.11) | 1.34 |
| **Total** |  | 111 (3.43) | 100 |

**Table S5**. Comparisons of dating from this study, Magallón et al. (2015), Grímsson et al. (2017) and Liu et al. (2018)

|  | **Magallón et al. (2015) (UCLN median) Ma** | **Grímsson et al. (2017) Rooting scenario 3 (UCLN median) Ma** | **Liu et al. (2018)**  **(UCLN median) Ma** | **This study (UCLN median) Ma** |
| --- | --- | --- | --- | --- |
| **Stem Loranthaceae** | 67.87 | N/A | 71.08 | 74.09 |
| **Crown Loranthaceae** | N/A | 50.8 | 59.38 | 62.77 |
| **Stem Scurrulinae** | N/A | 29.07 | 32.75 | 32.37 |
| **Crown Scurrulinae** | N/A | 17.96 | 19.93 | 25.46 |
| **Stem *Taxillus*** | N/A | 17.96 | 19.93 | 21.23 |
| **Stem *Scurrula*** | N/A | 17.96 | 19.93 | 21.23 |

**
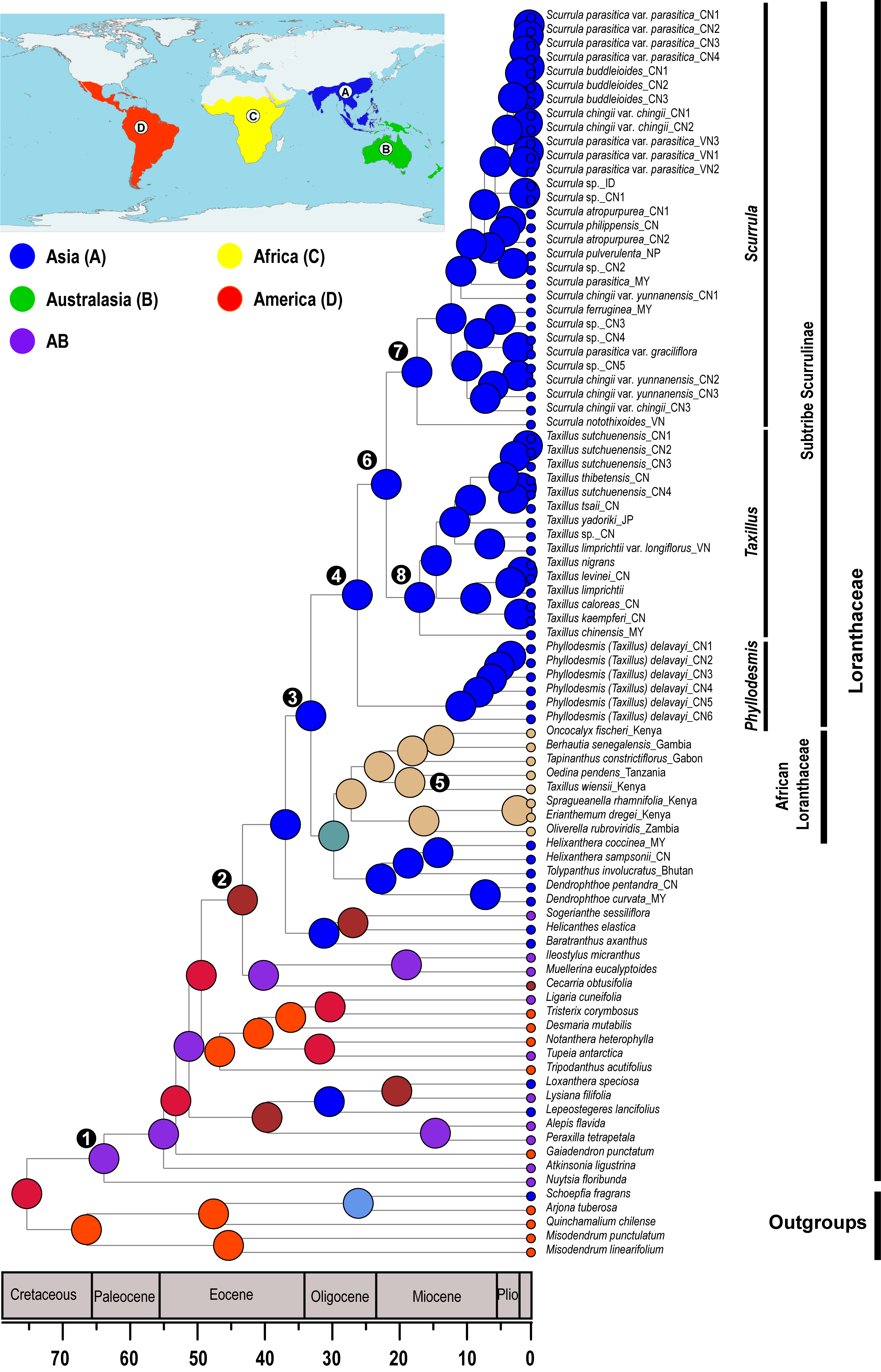
**

**Fig. S1.** Ancestral range reconstruction of Scurrulinae by Bayes-DIVA. Geologic time scale is shown at the bottom. Area abbreviations are as follows: A = Asia (including mainland of South Asia, Indochina and Malesia, but excluding New Guinea); B = Australasia (including Australia, New Zealand, New Guinea, and Pacific Islands); C = Africa (including the coastal area of the Arabian Peninsula and Sub-Saharan Africa); D = Americas (including Mexico, Central and South America). Abbreviations: CN: China, VN: Vietnam, ID: Indonesia; MY: Malaysia, JP: Japan, NP: Nepal.
